# Optimizing waiting experience: how passenger progress information and entertainment fillers jointly influence online ride-hailing drivers’ time perception and emotion

**DOI:** 10.1038/s41598-025-34811-9

**Published:** 2026-01-08

**Authors:** Yunguo Liu, Rang Meng, Xuelin Tang, Zizhen Liu

**Affiliations:** School of Arts, Chongqing University, Chongqing, China

**Keywords:** Time perception, Emotional experience, User experience, Interface icons, Entertainment fillers, Neuroscience, Psychology, Psychology

## Abstract

This study looked at how entertainment fillers and interface information display strategies affected drivers’ emotional experience and perceptions of time while waiting for passengers on online ride-hailing services. Three independent variables were used to complete the between-subjects experimental design: a countdown indicator (with or without), animated icons (pointer icon / humanoid icon) for passenger location, and entertainment fillers (with or without). The simulation experiment, which involved recruiting 50 skilled online ride-hailing drivers, assessed online ride-hailing drivers’ emotional experience and perceptions of time in great detail. The three-dimensional emotion model of pleasure-arousal-dominance and the theory of time perception were used to design the experiment. The results show that the countdown indicator and entertainment fillers significantly impact online ride-hailing drivers’ emotional experience and perceptions of time. According to the interaction impact between the countdown and the passenger animation symbols, the “humanoid icon” in the “with countdown” scenario results in a faster perception speed, but the “no countdown” situation has the reverse effect. The study’s results provide empirical evidence to enhance the waiting interface design for online ride-hailing rentals, especially when balancing functional indicators with cognitive loads.

## Introduction

With the rapid expansion of online ride-hailing services, drivers frequently experience passenger boarding waits^1^. Prolonged waiting creates a psychological burden and negative experiences^2^. As the primary interaction interface, waiting screen design significantly impacts driver experience^3^. Research shows that objective waiting time translates into differentiated perceived duration, with negative emotions positively correlating with this perception^4^. Interface fillers reduce waiting time estimate, indicating entertainment modes positively affect emotion^5^. Thus, optimizing interface design to minimize driver burnout is crucial^6^.In online ride-hailing ecosystems, drivers, passengers, and platforms connect through APP design^7,8^. Existing research primarily focuses on passenger satisfaction^8–10^ and service optimization^2,11^, while drivers’ situation remains understudied.

Despite being primary users, drivers’ waiting experience remains understudied^2^. Interface design significantly shapes driver experience^12^. Platforms employ multidimensional displays: dynamic countdowns^13^ and positioning animations^14^ enhance control by reducing information asymmetry. These elements interact to shape waiting perception^15–17^. Entertainment fillers substantially alter driver behavior^18^. While job complexity may compress subjective time^19^, whether entertainment fillers deplete attention and cause negative emotions requires verification. Thus, countdown indicators, passenger animations, and entertainment fillers in online ride-hailing contexts merit significant research attention.

Existing studies have found that countdowns improve time prediction accuracy^20^ and affect the user experience. Countdown prompts enhance excitement and alter time perception^21,22^. Well-designed icons reduce waiting boredom^23^, with studies confirming icons’ emotional impact through examinations of frequency^4,23^, type^4,16,24^, color^25^, and style^26^. Unoccupied waiting lengthens temporal perception^27^, while attention division improves waiting experience^2^.

Previous research reveals three persistent issues: Most current studies on online ride-hailing interfaces focus on passengers. The limited driver-related research primarily aims at service optimization, without genuinely addressing this group’s user experience. However, driver experience is crucial for team stability and platform sustainability within the expanding sharing economy. Second, while studies confirm that animated icons^4,16,25^, entertainment fillers^2,27^, and countdown indicator^13,20,21^ impact users’ time perception, emotional experience, and preference in waiting scenarios, most evidence comes from contexts like banking^28^, retail^23^, and pedestrian navigation^15^. The applicability of the existing theoretical framework to the group of online car-hailing drivers needs further validation and does not explain the interplay of interface factors in waiting situations for this particular group.

This study will be devoted to solving the above problems. Firstly, we theoretically apply the classical time perception theory and the PAD (Pleasure-Arousal-Dominance) three-dimensional emotion model to the design of the waiting interface in the mobile driving environment and verify their applicability in the group of online ride-hailing drivers. Secondly, we break through the limitation of previous interface design studies that focus on the main effect of a single factor and test the interaction effect between the countdown indicator, passenger animated icons, and entertainment fillers through a rigorous 2 × 2 × 2 experimental design. Finally, a structured user experience evaluation system is constructed by measuring multidimensional indicators of time perception and three dimensions of emotion. From a practical point of view, this study provides optimized design recommendations for the interface design of online ride-hailing platforms in the context of expanding the sharing economy and retaining the social well-being and concern for drivers, which is crucial for the platform’s sustainability.

## Related work

As digital interactions become increasingly prevalent, effective interface design is more critical than ever. In online ride-hailing services, drivers’ waiting experience is shaped by multiple factors, including countdown indicators, passenger animation icons, and entertainment fillers. While existing research on interface design has primarily focused on domains like entertainment, transportation, and shopping, significant gaps remain regarding online ride-hailing drivers’ waiting experience. This study addresses the overlooked driver perspective by examining three under-explored elements—countdown indicator, passenger animation icons, and entertainment fillers—and their interactions. Although academic research in this context remains sparse, substantial knowledge exists in broader user experience design. This paper first reviews literature on these three elements, establishes independent variables, then synthesizes interface user experience findings to define dependent variables, and finally outlines research objectives.

### Influencing factors

Research shows that progress indicators during system delays effectively enhance user performance. Kim’s study using a 7-point Likert scale demonstrated how loading symbol design affects waiting time perception^23^, while Lee found that color and information display in progress indicators influence user behavior on crowdfunding platforms^25^. Studies evaluating dimensions including shape^29^, speed^30^, direction^31^, and color^24^ consistently show that indicator variations elicit distinct user emotional experience and time perception^4^. As a typical progress indicator, countdown versions have received extensive attention. Li et al. found that countdown functionality yielded the shortest perceived waiting time in navigation apps^15^, while another study confirmed their superior perceived rationality and user preference^32^. Further research on direction, frequency, and speed has deepened understanding. Existing evidence indicates that the countdown progress indicator significantly impacts interface effectiveness, motivating this study to examine how driver-side countdown indicators affect time perception and user experience in online ride-hailing waiting scenarios.

As key interface elements, animated icons significantly shape user experience. Research shows that dynamic icons during waiting periods influence users’ time perception and emotional response. Harrison examined dynamic icons’ impact on waiting perception^3^; Thomaschke et al. found innovative loading animations improve perceived time and evoke positive emotions^4^. Regarding types, dynamic progress bars elicit more positive feelings than static ones^25^, while logo-type symbols perform better in time perception and experience evaluation^4^. In ride-hailing apps, passenger animated icons enhance drivers’ sense of control by conveying real-time location, reducing cognitive load, and improving emotional experience^4^. Different icon forms likely induce varied driver responses in time perception and emotional experience through distinct visual representations. This study thus incorporates passenger animated icons as an independent variable to examine their effects on users’ time perception, emotional experience, and preference.

Reducing users’ perceived wait time is a practical approach to enhance the waiting experience, where providing feedback helps users adapt to delays^30^. This implies that alleviating user anxiety during waiting can reduce subjective time perception. Borges et al. investigated how store TV screens distract and entertain waiting customers. Results showed that dynamic content captured attention, effectively reduced perceived wait time, and increased pleasure during waiting^33^. A related study comparing waiting conditions found the longest time perception in passive waiting, while inactive conditions reported the least enjoyment. Active waiting yielded the best outcomes when integrating time perception with emotional experience^30^. Thus, incorporating entertainment during waiting can substantially influence user experience, though this claim lacks thorough validation in the specific contexts examined in this study. Moreover, excessive entertainment or unsuitable tasks may deplete drivers’ attentional resources. This study examines whether providing entertainment content while waiting for passengers positively affects drivers’ mood and time perception.

### Temporal perception and emotional experience

Delays are prevalent in contemporary online contexts and adversely affect user perceptions and experiences^34^. Examining the online ride-hailing driver awaiting passengers’ situation, a comprehensive grasp of the factors influencing time perceptions and user experience can facilitate an exploration of users’ assessments of waiting. In this study, we used the theory of time perception and the PAD three-dimensional emotion model to look at how users feel about time, including how long they think they waited, how fast time seemed to pass, and how bored they felt, which are the four main ways to measure time perception. Additionally, the three-dimensional emotion model of pleasure-arousal-dominance was used to assess the user’s emotional experience, and a preference assessment was used to gather the user’s subjective preference.

The primary metrics for assessing subjective waiting experience are perceived duration, perceived pace, and perceived boredom^4^. Perceived duration refers to the subjective judgment of time intervals between successive events, influenced by sensory, attentional, emotional, and cognitive factors^25^, and closely correlates with user experience. Research shows that reducing perceived duration significantly enhances user experience, while prolonged perception negatively impacts interface experience^15^. The user’s perceived speed and boredom are equally crucial: faster perceived pace correlates with more positive experience and emotion^17^, while perceived boredom directly affects temporal perception and waiting experience evaluation^4^. Providing timely and innovative feedback during waits alleviates negative experience^16^ and diverts attention from waiting duration^35^. Consequently, this study employs perceived duration, pace, and reported boredom as indicators of subjective time perception.

An increasing number of product designers recognise that, in today’s information age, product designs must go beyond usability and simplicity to meet users’ emotional needs^4^. From a corporation’s standpoint, generating enhanced economic value necessitates considering the user’s emotional condition, as emotional needs are intricate and multifaceted^36^. Studies indicate that individuals in pleasant conditions see time as passing rapidly^2^. Simultaneously, both emotional valence and arousal influence temporal perception^37^. Consequently, this article incorporates the user’s emotional experience as a significant factor and references the PAD three-dimensional theory of emotion—pleasure, arousal, and dominance—as an evaluative framework. This model, introduced by Mehrabian and Russell in 1994, is a multidimensional measurement framework that offers a scientific and methodical approach to thoroughly evaluating user experience^38^.

### Relevant theory

The perception of time is a critical measurement in user experience^3,4,17^. According to the theory of time perception, an individual’s subjective time experience is not consistent with the objective physical time, and it is affected by both the external environment and personal cognition^19^. The influencing factors include sound, picture, temperature, etc. Therefore, many scholars have been working on the design of external factors to regulate the individual’s time perception. Thus, many scholars have researched from the perspective of external factors in designing and therefore regulating individual time perception. Some more mature theoretical models propose that when individuals devote more attentional resources to non-temporal external tasks or internal thinking, they pay less attention to temporal information, which leads to shorter subjective time assessment, perceived duration, and faster time perception. Conversely, attention to time increases when subjective temporal assessment and perception duration are lengthened, and time perception speed is slowed.

The PAD three-dimensional model of emotion was proposed by Mehrabian and Russell in 1974, which describes and measures emotion in three relatively independent dimensions: pleasantness, arousal, and dominance, thus capturing more complex and subtle changes in emotion^38^. The PAD model has now been widely validated in human-computer interaction and environmental psychology and is suitable for assessing users’ emotional responses in different physical and digital environments. The model has the advantage of combining the measurement of dominance, a measurement dimension that is crucial in uncertain waiting, with multidimensionality, so this model was chosen as the theoretical support for this study and applied to the specific digital environment of waiting for a online car, ensuring the comprehensiveness and theoretical depth of this study’s assessment of the user experience of online ride-hailing drivers.

### Research objectives

This study examines drivers’ experiences with three interface design characteristics through an in-depth analysis of the scenario involving an online ride-hailing driver awaiting a passenger. The research primarily addresses the subsequent enquiries:

1. How do countdown indications impact users’ perceptions of time, emotional experience, and subjective preference?
2. How do various passenger animation icons influence users’ perceptions of time, emotional experience, and subjective preference?
3. How do entertainment fillers impact users’ perception of time, emotional experience, and subjective preference?

## Materials and methods

### Experimental framework

**Fig. 1:**
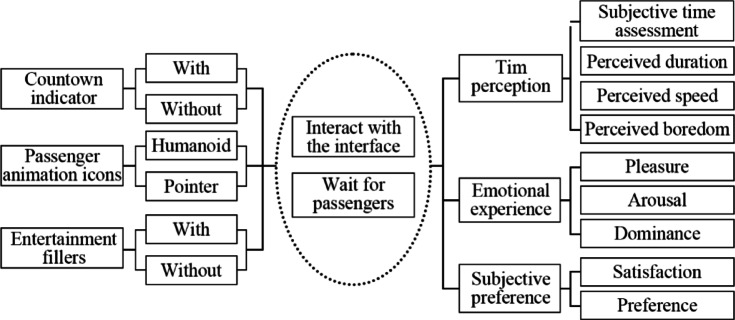
The research framework of this study.

In this study, a 2 (countdown indicator: with/without) x 2 (passenger animation icons: pointer icon/humanoid icon) x 2 (entertainment fillers: with entertainment function/without entertainment function) between-subjects experiment and subjective preference selection were used to collect data for analysis. Fig. 1 plots the study framework between the above three independent variables. The factors examined include time perception, emotional experience, and subjective preference, the basic metrics for measuring user experience in human-computer interaction. The experimental task in this study requires participating in operating a software prototype to simulate the order-taking process until waiting for passengers to board the bus is completed, and the mobile phone interface should be continuously observed during the waiting process.

The online taxi software employed in the experiment was crafted to imitate a virtual environment, altering experimental variables to assess participants’ time perception, emotional experience, and subjective preference. The experiment delineated a total of 9 measurement items.

The overall framework of this study is built based on time perception theory and the PAD three-dimensional emotion model. By setting three measurement indicators 1, 2, and 3, which constitute a multidimensional time perception measurement system, the time perception data of online ride-hailing drivers are comprehensively collected. Measurement indicators 4, 5, 6, and 7 are set according to the PAD three-dimensional emotion model, aiming to explore the user’s emotion data from a multidimensional perspective. Finally, two subjective questions 8 and 9 were added to measure the subjective preference of online ride-hailing drivers, as data collection from the subjective perspective of this study.

1. Subjective time assessment: Participants estimated the actual waiting duration in seconds by providing a precise figure ranging from 0 to 180 s (e.g., “120 seconds”).
2. Perceived duration: Participants’ comprehensive psychological evaluation of the waiting period, with endpoints designated “1 = long” to “7 = short”.
3. Perceived speed: Participants’ subjective assessment of the temporal pace experienced throughout the waiting period, with endpoints designated as “1 = slow” and “7 = fast”.
4. Perceived boredom: Participants evaluated their cognitive engagement and emotional experience during the waiting period, with endpoints ranging from “1 = boring” to “7 = interesting”.
5. Pleasure: A positive or negative inclination in participants’ affective valence during the waiting period, with endpoints designated “1 = sad” to “7 = happy”.
6. Arousal: Variation in the participant’s psychological arousal state, with endpoints designated “1 = calm” to “7 = excited”.
7. Dominance: Participants’ perception of control in the waiting scenario, rated from “1 = passive” to “7 = active”.
8. Satisfaction: Participants’ satisfaction with the design of the interface indicators in the experiment was measured on a scale from “1 = dissatisfied” to “5 = satisfied”.
9. Preference: Participants selected their favoured indicators based on subjective inclinations: countdown indicator (with / without), passenger animation symbols (pointer icon / humanoid icon), and entertainment fillers (with / without).

The measuring model breaks down how people perceive time into different factors and includes emotional and thinking aspects to create a structured way to evaluate how users experience waiting.

### Apparatus and prototype

**Fig. 2:**
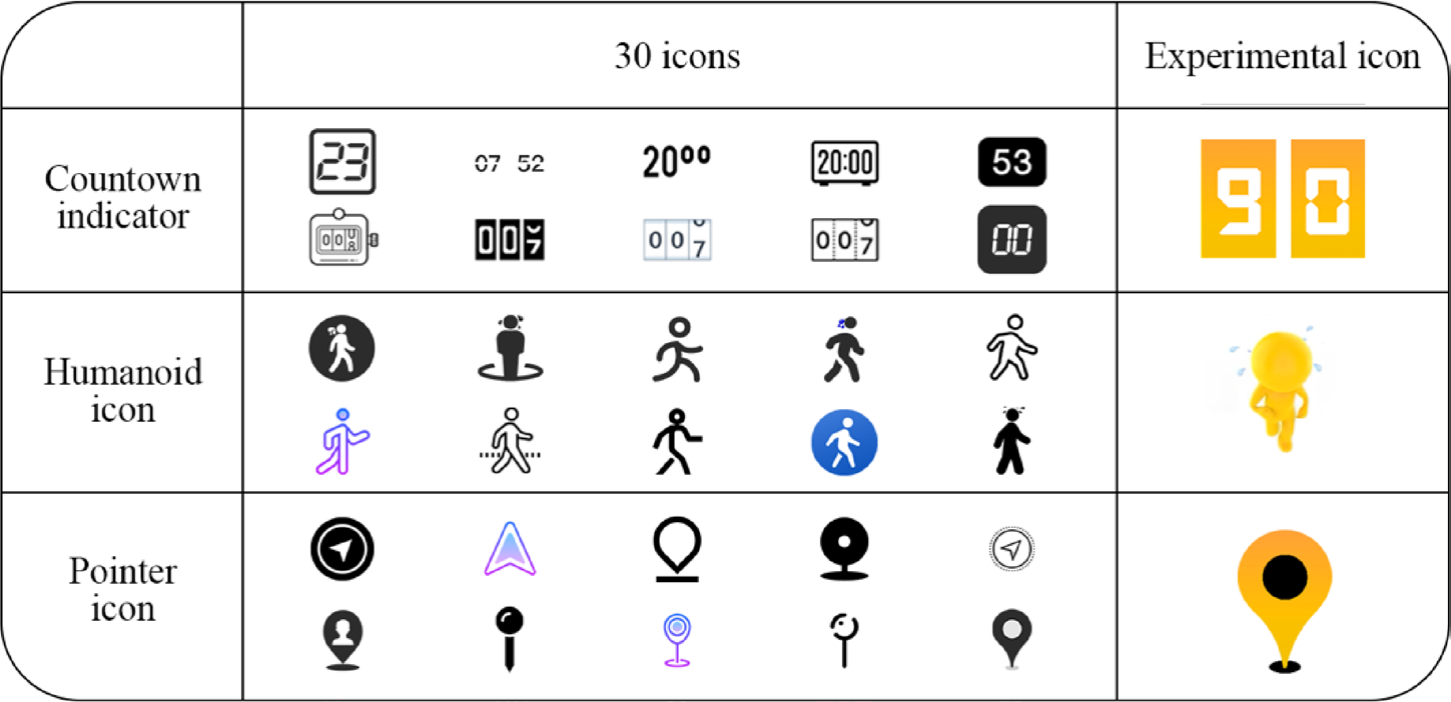
Experimental icon.

We thoroughly analyzed and ranked popular designer and icon design websites before the experiment. We selected ten websites to collect forty representative countdown icons and forty representative passenger animation icons. Subsequently, an expert group was convened to categorise the icons based on their attributes (countdown icons remain uncategorised, while passenger animation icons are divided into “pointer icon” and “humanoid icon”). Icons failing to meet the “countdown” criteria were discarded, resulting in ten countdown icons and ten passenger animation icons in each classification. In this experiment, the icon color scheme employs yellow as the foreground and accent color, paired with black and white as background and base colors. This approach comprehensively considers the current design landscape of ride-hailing platforms, visual cognitive requirements in driving environments, and principles of human factors engineering^39–41^. First, mainstream ride-hailing apps like Didi and Uber predominantly utilize high-contrast, low-saturation color schemes^39^. Theoretically, yellow optimizes visibility and alertness, effectively capturing attention without inducing anxiety^39,40^. The black-and-white contrast provides maximum brightness and color contrast, ensuring legibility under varying lighting conditions while meeting WCAG (Web Content Accessibility Guidelines) requirements for text-to-graphic contrast ratios^41^. This color strategy adheres to ISO 15008:2017 standards for human factors requirements in traffic information display systems^42^, emphasizing information clarity and comprehensibility while minimizing unnecessary visual distractions. A single representative countdown and two passenger animation icons have been produced according to their respective categories: “pointer icon” and “humanoid icon”, as illustrated in Fig. 2.

**Fig. 3:**
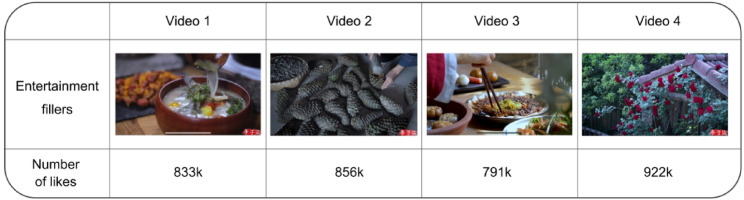
Entertainment fillers.

This study selects entertainment films for the experiment by employing material screening methods outlined in the literature^43^, which aim to eliminate any variability in the effects of entertainment fillers. Four films from notable creative bloggers were selected (as depicted in Fig. 3), demonstrating a uniform video style and identical content category. The four videos chosen for the control group were released within six months, with a narrow range of likes (mean = 850.5k; range = 71.5k) by selecting China’s largest short-video platform as the source of materials to guarantee the credibility and viewership of the data, regulating the number of likes on the chosen video materials to maintain fundamental consistency in public video preference, and ultimately ensuring that the release timing is proximate to uphold the basic uniformity of style and content across the various video materials.

**Fig. 4:**
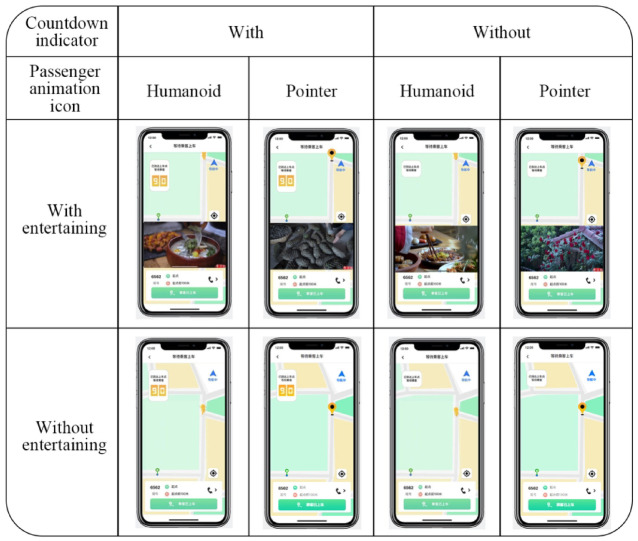
Experimental prototypes.

This study’s experimental design employs “Adobe After Effects CC” and “Mo Dao” software to facilitate the development of a mobile application prototype. The prototype is a mobile application designed to replicate the order-taking procedure from the driver’s perspective in an online ride-hailing service. The apparatus employed in the studies was a 6.1-inch iPhone 14 Pro featuring the iOS operating system, with a resolution of 2556 × 1179 pixels and a pixel density of 460 PPI. Eight distinct versions of the app prototype were developed, with various countdown indicators, passenger animation icons, and entertainment fillers (as illustrated in Fig. 4).The images displayed in Figs. 3 and 4 are sourced from publicly available resources on China’ s short-video platform.

### Participants

Fifty participants were randomly selected for the experiment using a purposive sampling technique. The experiment was performed outside on a secure street, involving one volunteer and two experimenters simultaneously. All participants were professional online ride-hailing drivers with experience utilising online ride-hailing software. All individuals exhibited proficient visual acuity and physical dexterity. Table 1 displays the demographics of the participants. 58% of the participants were under 40, while 44% were aged between 31 and 40.

Additionally, 4% identified as female and 96% as male. No participant experienced any fundamental operational issues during the experiment. The experiment duration was approximately 45 min, and participants received a remuneration of 50 RMB.

**Table 1:** Demographics of participants.

Division	Level	Frequency	Percentage (%)	Effective percentage (%)	Cumulative percentage (%)
Gender	Male	48	96.0	96.0	96.0
	Female	2	4.0	4.0	100.0
Age	21–30	7	14.0	14.0	14.0
	31–40	22	44.0	44.0	58.0
	41–50	13	26.0	26.0	84.0
	51–60	8	16.0	16.0	100.0
Driving experience	0–3	3	6.0	6.0	6.0
	4–5	4	8.0	8.0	14.0
	6–10	19	38.0	38.0	52.0
	More than 10	24	48.0	48.0	100.0
Education	Undergraduate and below	50	100.0	100.0	100.0

### Experimental procedure

Approval was obtained from the Institutional Review Board of Chongqing University[I20241205-R6]. We confirm that all research was performed per relevant guidelines/regulations. This study was reviewed and approved by an institutional review board (ethics committee) before the study began. Before the experiment commenced, the participants were given a thorough explanation of the informed consent form, and their written consent was acquired. A questionnaire was used to gather the experimental data, primarily distributed via the Questionnaire Star platform. The participants voluntarily completed the questionnaire after being told its purpose and that “submitting the questionnaire” constituted informed consent.

**Fig. 5:**
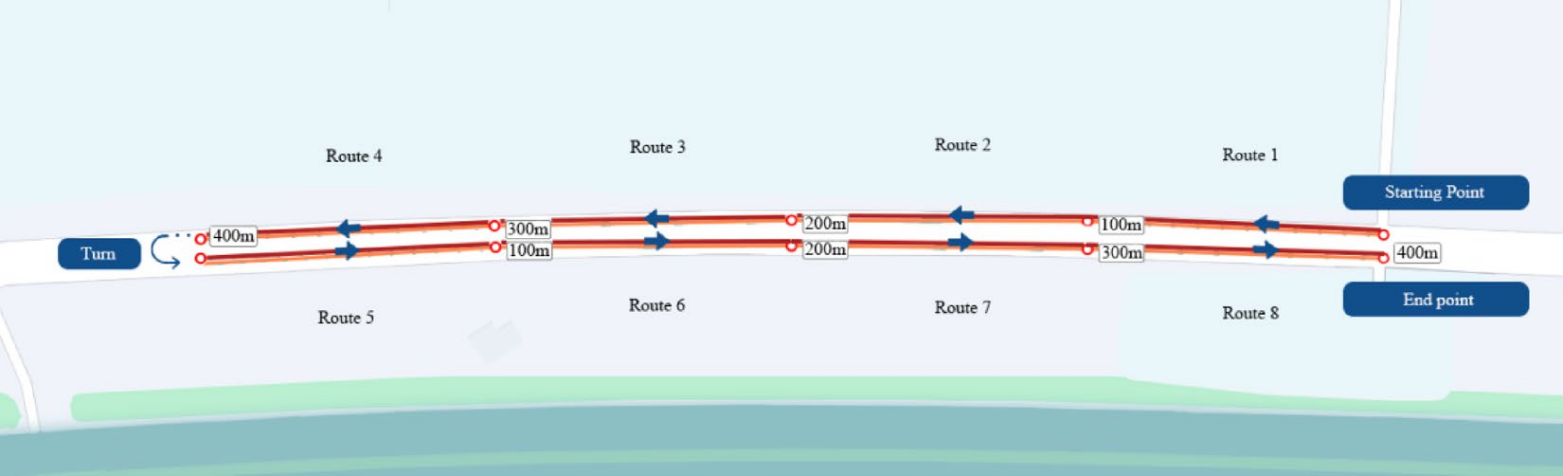
Experimental route.

**Fig. 6:**
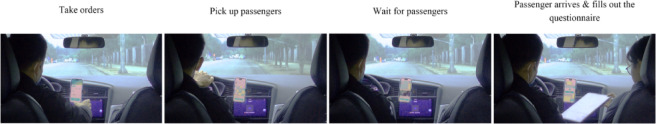
Experimental procedure.

This study simulates an online ride-hailing driver’s order-taking scenario in a secure outside roadway, ensuring no interference and maintaining consistent environmental variables (e.g., screen brightness, device model, etc.) throughout the process (as illustrated in Fig. 5). Each iteration of the 90-second waiting task included a real-time predictive countdown indicator, a pointer or humanoid icon denoting the passenger’s location, and a brief film for the entertainment group. To mitigate learning effects, each participant completed the entire experimental sample randomly, totalling eight rounds. Before the commencement of the trial, participants were obligated to complete a questionnaire that gathered fundamental information, such as age, gender, and driving experience. The experimenter thereafter familiarised the participants with the experimental process by guiding them through a prototype of the no-waiting stage interface. Participants were familiarised with the prototype by the experimenter, who provided a comprehensive explanation of the experimental tasks, which included taking the order, driving to the pickup location, waiting for the passenger to board the bus, and watching the interface(as illustrated in Fig. 6). Participants were randomly assigned to experience all eight prototypes at the beginning of the experiment. At the conclusion of each round, they were required to estimate the subjective waiting time and complete 7-point Likert scales reflecting their subjective time experience and the PAD three-dimensional affective model (refer to Table 2). After completing all experimental tasks, we asked participants to express their subjective preference through a questionnaire. The entire experiment lasted approximately 45 min.

**Table 2:** Survey questions.

Indicator	Item	Question
Temporal perception	Subjective time assessment	Estimated waiting time in seconds (60–180)
	Perceived duration	Long 1 2 3 4 5 6 7 short
	Perceived speed	Slow 1 2 3 4 5 6 7 fast.
	Perceived boredom	Boring 1 2 3 4 5 6 7 Interesting
Emotional experience	Pleasure	Sad 1 2 3 4 5 6 7 Happy
	Arousal	Calm 1 2 3 4 5 6 7 Excited
	Dominance	Passive 1 2 3 4 5 6 7 Active
Subjective preference	Satisfaction	Dissatisfied 1 2 3 4 5 Satisfied
	Preference	Favorite types of indicators

## Results

This study employed a 2 × 2 × 2 mixed factorial design, with all independent factors (countdown indicator, passenger animation icons, and entertainment fillers) classified as within-subject variables. The impact of countdown indicator, passenger animation icons, entertainment fillers, and their interactions on participants’ perceptions of time, emotional experience, and subjective preference was analysed using SPSS software. We subsequently used LSD post hoc tests to analyse significant main effects and elucidate variations among the component levels. The analysis of subjective preference focused on participants’ estimations of time and their preference for various types within the relevant variables.

Table 3 presents the comprehensive mean and standard deviation data, demonstrating that the “countdown indicator” enhances time perceptions, emotional experience, and contentment. Secondly, passenger animation symbols in the “humanoid icon” will improve users’ perceptions of time, emotional experience, and overall happiness, resulting in lower subjective length estimations. Consequently, offering passenger animation icons, including the “humanoid icon”, will enhance the user experience. The influence of the two categories of entertainment fillers on participants was markedly distinct. The “with entertainment” category markedly diminishes the perception of time for users while enhancing emotional experience and enjoyment. Furthermore, “with entertainment” also indicated diminished values regarding subjective duration assessments.

**Table 3:** Mean and standard deviation results for different countdown indicators, passenger animation icons, and entertainment fillers.

Variant	Countdown indicator				Passenger animation icons				Entertainment fillers			
	With		Without		Humanoid icon		Pointer icon		With		Without	
	M	SD	M	SD	M	SD	M	SD	M	SD	M	SD
Subjective time assessment	75.02	30.539	74.55	37.836	74.71	34.715	74.85	34.046	66.79	30.611	82.78	36.037
Perceived duration	4.46	1.917	4.04	2.147	4.36	2.110	4.13	1.974	5.39	1.506	3.11	1.872
Perceived speed	4.18	1.987	3.67	2.072	4.00	2.076	3.84	2.013	5.00	1.798	2.84	1.670
Perceived boredom	4.06	2.133	3.69	2.197	3.96	2.182	3.79	2.161	5.19	1.777	2.56	1.685
Pleasure	4.54	1.683	4.21	1.805	4.49	1.742	4.25	1.756	5.23	1.532	3.51	1.527
Arousal	3.77	1.851	3.36	1.799	3.68	1.870	3.46	1.796	4.20	1.915	2.93	1.505
Dominance	4.30	1.916	3.50	1.934	4.03	2.007	3.77	1.917	4.75	1.754	3.05	1.797
Satisfaction	3.54	1.164	3.13	1.326	3.46	1.235	3.20	1.281	3.93	1.008	2.74	1.209

### Time perception measurement

#### Subjective time assessment

Subjective time evaluations comprised participants’ predictions of waiting durations; their exact subjective waiting periods were recorded in seconds (s).

The within-subject ANOVA results in Table 4 indicate that the countdown indicator category’s main effect was insignificant. The passenger animation icons category exhibited no statistically significant main impact (F = 0.002, *p* = 0.986 > 0.05; $\:{\eta\:}_{p}^{2}$ < 0.001). A notable main effect was observed for the entertainment fillers category (F = 22.644, *p* < 0.001; $\:{\eta\:}_{p}^{2}$ = 0.055). The LSD posttest findings indicated a substantial difference between conditions “without entertainment” and “with entertainment”. In general, participants in the “with entertainment” group reported shorter subjective time assessments (M = 66.79, SD = 30.611) compared to those in the “without entertainment” condition (M = 82.78, SD = 36.037). This data indicates that the “with entertainment” condition markedly diminished participants’ subjective estimations of waiting time.

**Table 4:** Subjective time assessment: within-subjects ANOVA.

Source	Implicit variable	SS	DF	MS	F	*p*	$\:{\eta\:}_{p}^{2}$	Post Hoc (HSD)
Countdown indicator	Subjective time assessment	21.622	1	21.622	0.019	0.890	< 0.001	
Passenger animation icons	Subjective time assessment	1.823	1	1.823	0.002	0.968	< 0.001	
Entertainment fillers	Subjective time assessment	25584.003	1	25584.003	22.644	< 0.001	0.055	With Entertainment > Without Entertainment
Countdown indicator * passenger animation icons	Subjective time assessment	336.723	1	336.723	0.298	0.585	0.001	
Countdown indicator * entertainment fillers	Subjective time assessment	742.563	1	742.563	0.657	0.418	0.002	
Passenger animation icon * entertainment fillers	Subjective time assessment	39.063	1	39.063	0.035	0.853	< 0.001	
Countdown indicator * passenger animation icons * entertainment fillers	Subjective time assessment	861.423	1	861.423	0.762	0.383	0.002	

Additionally, there was no statistically significant interaction between the countdown indication and passenger animation icons (F = 0.298, *p* = 0.585 > 0.05; $\:{\eta\:}_{p}^{2}$ = 0.001). No substantial difference in the interaction between the countdown indication and entertainment fillers (F = 0.657, *p* = 0.418 > 0.05; $\:{\eta\:}_{p}^{2}$ = 0.002). Passenger animation icons and entertainment fillers do not significantly alter their interaction (F = 0.035, *p* = 0.853 > 0.05; $\:{\eta\:}_{p}^{2}$ < 0.001). Ultimately, there is no substantial change in the interaction among the countdown indicator, passenger animation icons, and entertainment fillers (F = 0.762, *p* = 0.383 > 0.05; $\:{\eta\:}_{p}^{2}$ = 0.002).

#### Perceived duration

Perceived duration refers to the time interval recognised by the participant based on their subjective experience, namely the individual’s own awareness of the duration.

Table 5 shows the results of the two-way ANOVA based on how people perceive time, focusing on the effects of the countdown indicator, passenger animation icons, and entertainment fillers. The primary impact of the countdown indication showed significant differences (F = 6.028, *p* = 0.015 < 0.05; $\:{\eta\:}_{p}^{2}$ = 0.015). The LSD posttest results indicate that the “With countdown” and “Without countdown” kinds exhibit significant differences (*p* = 0.015 < 0.05). Participants in the “with countdown” condition (M = 4.46, SD = 1.917) perceived shorter durations than those in the “without countdown” condition (M = 4.04, SD = 2.147). No substantial change was observed in the primary effect of passenger animation icons (F = 1.772, *p* = 0.184 > 0.05; $\:{\eta\:}_{p}^{2}$ = 0.012). A big difference was found for the main effect of entertainment fillers (F = 181.163, *p* < 0.001;$\:{\eta}_{\mathrm{p}}^{\mathrm{2}}$ = 0.036), and further testing showed a clear difference between the “with entertainment” and “without entertainment” conditions(*p* < 0.001). The perceived time was shorter for participants in the “with entertainment” condition (M = 5.39, SD = 1.506) compared to the “without entertainment” condition (M = 3.11, SD = 1.872).

**Table 5:** Time perception: within-subject ANOVA.

Source	Implicit variable	SS	DF	MS	F	*p*	$\:{\eta\:}_{p}^{2}$	Post Hoc (HSD)
Countdown indicator	Perceived duration	17.223	1	17.223	6.028	0.015	0.015	With Countdown > Without Countdown
	Perceived speed	26.523	1	26.523	9.065	0.003	0.023	With Countdown > Without Countdown
	Perceived boredom	13.690	1	13.690	4.607	0.032	0.012	With Countdown > Without Countdown
Passenger animation icons	Perceived duration	5.063	1	5.063	1.772	0.184	0.012	
	Perceived speed	2.403	1	2.403	0.821	0.365	0.014	
	Perceived boredom	2.890	1	2.890	0.972	0.325	0.053	
Entertainment fillers	Perceived duration	517.563	1	517.563	181.163	< 0.001	0.036	With entertainment > Without entertainment
	Perceived speed	468.722	1	468.722	160.205	< 0.001	0.005	With entertainment > Without entertainment
	Perceived boredom	686.440	1	686.440	230.982	< 0.001	0.002	With entertainment > Without entertainment
Countdown indicator * passenger animation icons	Perceived duration	4.203	1	4.203	1.471	0.226	0.002	
	Perceived speed	18.063	1	18.063	6.174	0.013	0.006	
	Perceived boredom	2.890	1	2.890	0.972	0.325	0.004	
Countdown indicator * entertainment fillers	Perceived duration	0.723	1	0.723	0.253	0.615	0.006	
	Perceived speed	0.563	1	0.563	0.192	0.661	0.014	
	Perceived boredom	4.000	1	4.000	1.346	0.247	0.316	
Passenger animation icons * entertainment fillers	Perceived duration	0.723	1	0.723	0.253	0.615	0.290	
	Perceived speed	0.003	1	0.003	0.001	0.977	0.371	
	Perceived boredom	4.840	1	4.840	1.629	0.203	0.244	
Countdown indicator * passenger animation icons * entertainment fillers	Perceived duration	1.103	1	1.103	0.386	0.535	0.123	
	Perceived speed	3.423	1	3.423	1.170	0.280	0.196	
	Perceived boredom	0.040	1	0.040	0.013	0.908	0.236	

There was no statistically significant interaction between the countdown indication and passenger animation icons (F = 1.471, *p* = 0.226 > 0.05; $\:{\eta}_{\mathrm{p}}^{\mathrm{2}}$ = 0.002). The countdown indication and entertainment fillers did not significantly differ in their interaction (F = 0.253, *p* = 0.615 > 0.05; $\:{\eta\:}_{p}^{2}$ = 0.006). Passenger animation icons and entertainment fillers do not significantly differ in their interaction (F = 0.253, *p* = 0.615 > 0.05; $\:{\eta\:}_{p}^{2}$ = 0.290). Ultimately, there is no substantial change in the interaction among the countdown indication, passenger animation icons, and entertainment fillers (F = 0.386, *p* = 0.535 > 0.05; $\:{\eta\:}_{p}^{2}$ = 0.123).

#### Perceived speed

Perceived speed denotes participants’ subjective assessment of the rate at which they perceive time to elapse. It represents the participant’s subjective assessment of the perceived passage of time, influenced by their emotions.

The results in Table 5 show that the countdown indicator had a significant impact (F = 9.065, *p* = 0.003 < 0.01; $\:{\eta\:}_{p}^{2}$ = 0.023). The LSD posttest indicated a significant difference between the “with countdown” and “without countdown” conditions (*p* = 0.003<0.01). Additionally, participants assessed the countdown as more rapid in the “with countdown” condition (M = 4.18, SD = 1.987) and as more sluggish in the “without countdown” condition (M = 3.67, SD = 2.072). No significant differences were observed for the primary effect of passenger animation icons (F = 0.821, *p* = 0.365 > 0.05; $\:{\eta\:}_{p}^{2}$ = 0.014). The main effect of entertainment fillers was significant (F = 160.205, *p* < 0.001; $\:{\eta\:}_{p}^{2}$ = 0.005), and there was a noticeable difference between the “with entertainment” and “without entertainment” groups in the LSD posttest (*p* < 0.001). Furthermore, participants in the “with entertainment” condition (M = 5.00, SD = 1.798) perceived the entertainment more rapidly than those in the “without entertainment” condition (M = 2.84, SD = 1.670).

**Fig. 7:**
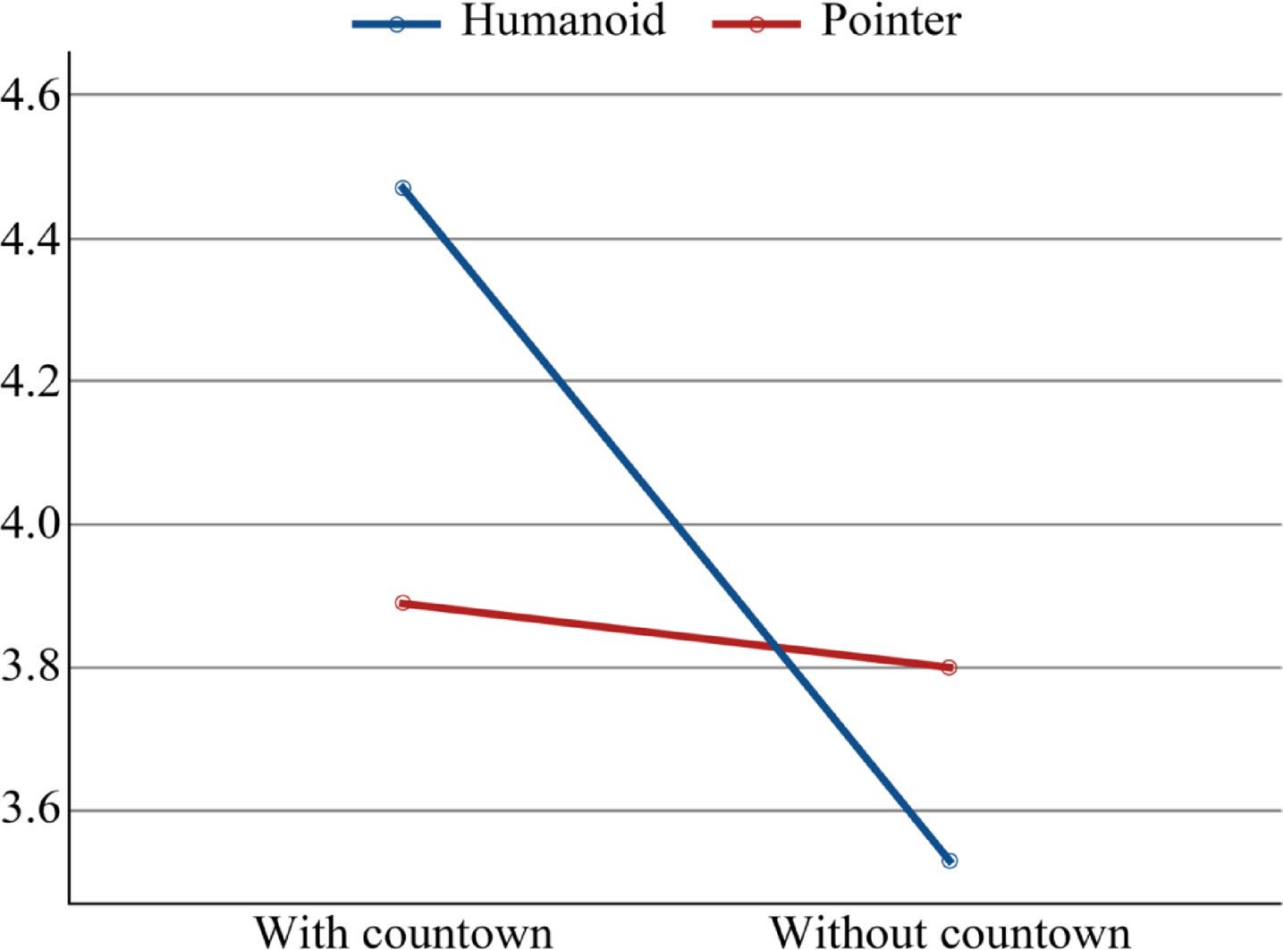
Interaction diagram between the countdown of perceived speed and the passenger metaphor.

Notably, a substantial difference exists in the interaction between the countdown indication and passenger animation icons (F = 6.174, *p* = 0.013 < 0.05; $\:{\eta\:}_{p}^{2}$ = 0.006). As illustrated in Fig. 7, under the condition of “with the countdown”, participants recognised the passenger pointer of the “humanoid icon” considerably more swiftly than the passenger animation symbol of the “pointer icon”. The contrary outcome was noted in the “without countdown” condition, where the “pointer icon” type of passenger animation icons was recognised more swiftly than the “humanoid icon” of the passenger animation icons. The perceived speed in the “with countdown” scenario is often greater than in the “without countdown” scenario, and a countdown markedly influences the perceived speed. There was no significant difference in the interaction between the countdown indication and entertainment fillers (F = 0.192, *p* = 0.661 > 0.05; $\:{\eta\:}_{p}^{2}$ = 0.014). The interaction between passenger animation icons and entertainment fillers is not significantly different (F = 0.001, *p* = 0.977 > 0.05; $\:{\eta\:}_{p}^{2}$ = 0.371). Ultimately, there is no substantial change in the interaction among the countdown indicator, passenger animation icons, and entertainment fillers (F = 1.170, *p* = 0.280 > 0.05; $\:{\eta\:}_{p}^{2}$ = 0.196).

#### Perceived boredom

If an individual perceives a lack of engaging information while waiting, boredom may arise, commonly referred to as feeling bored.

Based on the results generated in Table 5 regarding the relationship between the countdown indicator, passenger animation icons, and entertainment fillers, the main effect of the countdown indicator was found to be statistically significant (F = 4.607, *p* = 0.032 < 0.05; $\:{\eta\:}_{p}^{2}$ =0.012). The results of the post-test showed that there was a statistically significant main effect between the types of “with countdown” and “without countdown” shows, significant differences (*p* = 0.032 < 0.05). According to Table 3, participants in the “with countdown” condition (M = 4.06, SD = 2.133) had less boredom than those in the “without countdown” condition (M = 3.69, SD = 2.197). Furthermore, there was no notable change in the primary effect of the passenger animation symbol (F = 0.972, *p* = 0.325 > 0.05; $\:{\eta\:}_{p}^{2}$ = 0.053). A significant difference was found for the main effect of entertainment fillers (F = 230.982, *p* < 0.001; $\:{\eta\:}_{p}^{2}$ = 0.002), and the LSD post test showed a significant difference between “with entertainment” and “without entertainment” types (*p* < 0.001). The perceived boredom of participants in the “with entertainment” condition (M = 5.19, SD = 1.777) was substantially lower than that of those in the “without entertainment” condition (M = 2.56, SD = 1.685). Evidently, “with entertainment” markedly diminished participants’ subjective boredom throughout the waiting interval. The interaction between the countdown indication and passenger animation icons (F = 0.972, *p* = 0.325 > 0.05; $\:{\eta\:}_{p}^{2}$ = 0.004) was not statistically significant.

Furthermore, there was no notable change in the interaction between the countdown indication and entertainment fillers (F = 1.346, *p* = 0.247 > 0.05; $\:{\eta\:}_{p}^{2}$ = 0.316). No substantial difference was observed in the interaction between passenger animation icons and entertainment fillers (F = 1.629, *p* = 0.203 > 0.05; $\:{\eta\:}_{p}^{2}$ = 0.244). Ultimately, there is no substantial change in the interaction among the countdown indicator, passenger animation icons, and entertainment fillers (F = 0.13, *p* = 0.908 > 0.05; $\:{\eta\:}_{p}^{2}$ = 0.236).

### Emotional experience measurement

#### Pleasure

Pleasure was characterised by the subject’s emotional state (positive or negative) during the waiting period.

The results in Table 6 show that the countdown indicator had a significant effect (F = 4.713, *p* = 0.031 < 0.05; $\:{\eta\:}_{p}^{2}$ = 0.012). The LSD posttest indicated a significant difference between the “with countdown” and “without countdown” conditions (*p* = 0.031). Participants in the “with countdown” condition (M = 4.54, SD = 1.683) reported greater enjoyment than those in the “without countdown” condition (M = 4.21, SD = 1.805). No substantial variations were observed for the primary effect of passenger animation icons (F = 2.493, *p* = 0.115 > 0.05; $\:{\eta\:}_{p}^{2}$ = 0.006). The main effect of entertainment fillers was significant (F = 126.551, *p* < 0.001; $\:{\eta\:}_{p}^{2}$=0.244), and follow-up tests showed a big difference between the “with entertainment” and “without entertainment” groups (*p* < 0.001). Moreover, participants in the “with entertainment” condition (M = 5.23, SD = 1.532) found the experience more enjoyable than those in the “without entertainment” condition (M = 3.51, SD = 1.527).

**Table 6:** Emotional experience: within-subject ANOVA.

Source	Implicit variable	SS	DF	MS	F	*p*	$\:{\eta\:}_{p}^{2}$	Post Hoc (HSD)
Countdown indicator	Pleasure	10.890	1	10.890	4.713	0.031	0.012	With Countdown > Without Countdown
	Arousal	16.000	1	16.000	5.443	0.020	0.014	With Countdown > Without Countdown
	Dominance	65.610	1	65.610	21.900	< 0.001	0.053	With Countdown > Without Countdown
Passenger animation icons	Pleasure	5.760	1	5.760	2.493	0.115	0.006	
	Arousal	4.840	1	4.840	1.647	0.200	0.004	
	Dominance	6.760	1	6.760	2.256	0.134	0.006	
Entertainment fillers	Pleasure	292.410	1	292.410	126.551	< 0.001	0.244	With entertainment > Without entertainment
	Arousal	161.290	1	161.290	54.870	< 0.001	0.123	With entertainment > Without entertainment
	Dominance	285.610	1	285.610	95.333	< 0.001	0.196	With entertainment > Without entertainment
Countdown indicator * passenger animation icons	Pleasure	5.290	1	5.290	2.289	0.131	0.006	
	Arousal	5.290	1	5.290	1.800	0.181	0.005	
	Dominance	2.250	1	2.250	0.751	0.387	0.002	
Countdown indicator * entertainment fillers	Pleasure	< 0.001	1	0.000	< 0.001	1.000	< 0.001	
	Arousal	1.960	1	1.960	0.667	0.415	0.002	
	Dominance	0.160	1	0.160	0.053	0.817	< 0.001	
Passenger animation icons * entertainment fillers	Pleasure	1.690	1	1.690	0.731	0.393	0.002	
	Arousal	0.160	1	0.160	0.054	0.816	< 0.001	
	Dominance	1.210	1	1.210	0.404	0.525	0.001	
Countdown indicator * passenger animation icons * entertainment fillers	Pleasure	1.440	1	1.440	0.623	0.430	0.002	
	Arousal	0.490	1	0.490	0.167	0.683	< 0.001	
	Dominance	4.000	1	4.000	1.335	0.249	0.003	

There was no statistically significant interaction between the countdown indication and passenger animation icons (F = 2.289, *p* = 0.131 > 0.05; $\:{\eta\:}_{p}^{2}$ = 0.006). No substantial difference was observed in the interaction between the countdown indication and entertainment fillers (F < 0.001, *p* = 1.000 > 0.05; $\:{\eta\:}_{p}^{2}$ < 0.001). There is no substantial difference in the interaction between passenger animation icons and entertainment fillers (F = 0.731, *p* = 0.393 > 0.05; $\:{\eta\:}_{p}^{2}$ = 0.002). Ultimately, there is no substantial change in the interaction among the countdown indicator, passenger animation icons, and entertainment fillers (F = 0.623, *p* = 0.430 > 0.05; $\:{\eta\:}_{p}^{2}$ = 0.002).

#### Arousal

Arousal denotes the user’s mental state as either positive or negative.

The results in Table 6 show that the countdown indicator had a significant impact (F = 5.443, *p* = 0.020 < 0.05; $\:{\eta\:}_{p}^{2}$=0.014). The LSD posttest findings indicated a significant difference between the “with countdown” and “without countdown” conditions (*p* = 0.020 < 0.05). Participants in the “with countdown” condition (M = 3.77, SD = 1.851) exhibited greater positivity than those in the “without countdown” condition (M = 3.36, SD = 1.799). The primary impact of passenger motion icons was not statistically significant (F = 1.647, *p* = 0.200 > 0.05; $\:{\eta\:}_{p}^{2}$ = 0.004). The main effect of entertainment fillers was significant (F = 54.870, *p* < 0.001; $\:{\eta\:}_{p}^{2}$ = 0.123), and follow-up tests showed a clear difference between the groups that had entertainment and those that did not (*p* < 0.001). Furthermore, participants in the “with entertainment” condition (M = 4.20, SD = 1.915) exhibited greater positive motivation than those in the “without entertainment” condition (M = 2.93, SD = 1.505).

There was no statistically significant interaction between the countdown indication and passenger animation icons (F = 1.800, *p* = 0.181 > 0.05; $\:{\eta\:}_{p}^{2}$ = 0.005). No substantial change was seen in the interaction between the countdown indication and entertainment fillers (F < 0.667, *p* = 0.415 > 0.05; $\:{\eta\:}_{p}^{2}$ = 0.002). Additionally, there was no significant difference in the interaction between passenger animation icons and entertainment fillers (F = 0.054, *p* = 0.816 > 0.05; $\:{\eta\:}_{p}^{2}$ < 0.001). The interaction between the countdown indication, passenger animation icons, and entertainment fillers ultimately does not significantly change (F = 0.167, *p* = 0.683 > 0.05; $\:{\eta\:}_{p}^{2}$ < 0.001).

#### Dominance

Dominance refers to the dominant feelings of the subjects during the waiting period: passive waiting and having a sense of control, respectively.

Results from a two-way ANOVA indicated a significant difference in the primary effect of the countdown indicator (F = 21.900, *p* < 0.001; $\:{\eta\:}_{p}^{2}$ = 0.053). The LSD posttest indicated a statistically significant difference between the “with countdown” and “without countdown” conditions (*p* < 0.001). According to Table 3, participants exhibited a heightened sense of control in the “with countdown” condition (M = 4.30, SD = 1.916) compared to the “without countdown” condition (M = 3.50, SD = 1.934). No substantial variations were observed for the primary effect of passenger animation icons (F = 2.256, *p* = 0.134 > 0.05; $\:{\eta\:}_{p}^{2}$ = 0.006). The main effect of entertainment fillers was significant (F = 95.333, *p* < 0.001; $\:{\eta\:}_{p}^{2}$ = 0.196), and follow-up tests showed a big difference between the groups with and without entertainment (*p* < 0.001). Furthermore, individuals in the “with entertainment” group (M = 4.75, SD = 1.754) experienced a greater sense of control compared to those in the “without entertainment” condition (M = 3.05, SD = 1.797).

There was no statistically significant interaction between the countdown indication and passenger animation icons (F = 0.751, *p* = 0.387 > 0.05; $\:{\eta\:}_{p}^{2}$ = 0.002). The countdown indication and entertainment fillers do not significantly differ in their interaction (F = 0.053, *p* = 0.817 > 0.05; $\:{\eta\:}_{p}^{2}$ < 0.001). Passenger animation icons and entertainment fillers do not significantly differ in their interaction (F = 0.404, *p* = 0.525 > 0.05; $\:{\eta\:}_{p}^{2}$ = 0.001). Ultimately, there is no substantial change in the interaction among the countdown indicator, passenger animation icons, and entertainment fillers (F = 0.1335, *p* = 0.249 > 0.05; $\:{\eta\:}_{p}^{2}$ = 0.003).

### Subjective preference measurement

#### Satisfaction

Satisfaction indicates whether the experimental participants had a good experience and were satisfied with the content of the measurements.

The results in Table 7 show that the countdown indicator had a big effect, with a significant difference (F = 14.827, *p* < 0.001; $\:{\eta\:}_{p}^{2}$ = 0.036). The LSD posttest indicated a statistically significant difference between the “with countdown” and “without countdown” conditions (*p* < 0.001). Participants in the “with countdown” condition (M = 3.54, SD = 1.164) exhibited greater satisfaction than those in the “without countdown” condition (M = 3.13, SD = 1.326). A significant difference was found in how passenger animation icons affected satisfaction (F = 5.682, *p* = 0.018 < 0.05; $\:{\eta\:}_{p}^{2}$ = 0.014), with the LSD post-hoc test showing a meaningful difference between “humanoid icon” and “pointer icon” (*p* = 0.018 < 0.05). Participant satisfaction was greater in the “humanoid icons” condition (M = 3.46, SD = 1.235) compared to the “pointer icon” condition (M = 3.20, SD = 1.281). The effect of entertainment fillers was significant (F = 121.040, *p* < 0.001; $\:{\eta\:}_{p}^{2}$ = 0.236), showing a clear difference between the “with entertainment” and “without entertainment” groups (*p* < 0.001), where those with entertainment were much more satisfied. Participants in the “with entertainment” condition (M = 3.93, SD = 1.008) exhibited more satisfaction than those in the “without entertainment” condition (M = 2.74, SD = 1.209).

**Table 7:** Satisfaction: within-subject ANOVA.

Source	Implicit variable	SS	DF	MS	F	*p*	$\:{\eta\:}_{p}^{2}$	Post Hoc (HSD)
Countdown indicator	Satisfaction	17.640	1	17.640	14.827	< 0.001	0.036	With Countdown > Without Countdown
Passenger animation icons	Satisfaction	6.760	1	6.760	5.682	0.018	0.014	Humanoid > Pointers
Entertainment fillers	Satisfaction	144.000	1	144.000	121.040	< 0.001	0.236	With entertainment > Without entertainment
Passenger animation icons	Satisfaction	0.250	1	0.250	0.210	0.647	0.001	
Countdown * entertainment fillers	Satisfaction	1.210	1	1.210	1.017	0.314	0.003	
Passenger animation icons * entertainment fillers	Satisfaction	0.250	1	0.250	0.210	0.647	0.001	
Entertainment fillers	Satisfaction	0.640	1	0.640	0.538	0.464	0.001	

There was no statistically significant interaction between countdown and passenger animation icons (F = 0.210, *p* = 0.647 > 0.05; $\:{\eta\:}_{p}^{2}$ = 0.001). There is no significant difference in the interaction between countdown and entertainment fillers (F = 1.017, *p* = 0.314 > 0.05; $\:{\eta\:}_{p}^{2}$ = 0.003). No substantial difference exists in the interaction between passenger animation icons and entertainment fillers (F = 0.210, *p* = 0.647 > 0.05; $\:{\eta\:}_{p}^{2}$ = 0.001). Ultimately, there is no substantial change in the interaction among countdown, passenger animation icons, and entertainment fillers (F = 0.538, *p* = 0.464 > 0.05; $\:{\eta\:}_{p}^{2}$ = 0.001).

#### Preference

Preference selections indicated respondents’ tendencies and encompassed preferred countdown indication types, passenger animation symbol types, and entertainment filler types.

**Fig. 8:**
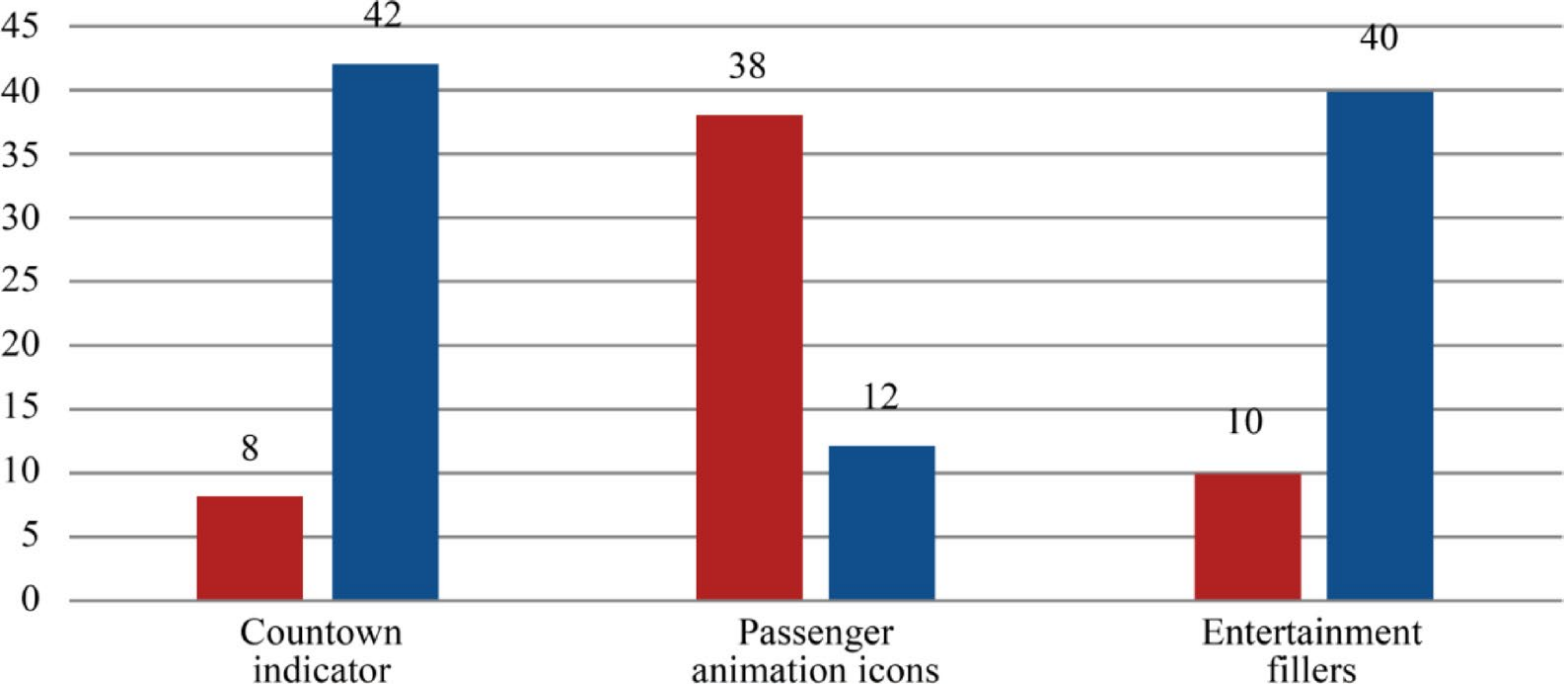
Preference statistics of subjects.

As indicated by the subjective preference data in Fig. 8, merely 16% of the participants selected “without countdown” for the countdown indicator, whereas 84% opted for “with countdown”. A greater number of subjects favoured the “with countdown” group. Of the categories of passenger animation icons, 76% of participants selected “humanoid icon”, while 24% opted for “pointer icon”. A greater number of respondents favoured the passenger animation icons in the “humanoid icon” category over those in the “pointer icon” category. Ultimately, 20% selected “without entertainment”, while 80% opted for “with entertainment”. A greater number of subjects chose the “with entertainment” category.

## Discussion and implications

### Summary of findings

The results indicated that only entertainment fillers influenced users’ subjective time perception, with participants estimating a considerably shorter duration for the “with entertainment” genre, aligning with other research findings^2^. Furthermore, the primary impacts of perceived duration, perceived speed, and reported boredom yielded identical outcomes. Participants believed that the “with the countdown” genre contributed to a reduced perception of duration, heightened enjoyment while waiting, and an increased sense of speed, as corroborated by data from other research^4,44^. The interaction impact of perceived speed reveals that “humanoid icon” are generally viewed as faster than “pointer icon” in the “with countdown” scenario. In the “without a countdown” condition, participants viewed the “pointer icon” to convey a faster perceived pace than the “humanoid icon”. The primary effect research on time perception revealed no statistical differences among passenger animation icons.

The findings indicate that passenger animation icons do not significantly influence users’ emotions. This conclusion contradicts the results of certain prior studies suggesting that animated icons can enhance user pleasantness^4^. The primary effects of pleasure, arousal, and dominance demonstrated consistent outcomes for both the countdown indication and entertainment fillers. The “with countdown” and “with entertainment” categories generally offered a more pleasurable and stimulating experience than the control group. Moreover, the “with countdown” and “with entertainment” categories gave participants greater control over the experience. This conclusion indicates that in the absence of counting and entertainment, participants’ enjoyment diminishes, and their emotions gradually stabilise, leading to a sense of reduced control.

The satisfaction survey results indicated that participants expressed greater satisfaction with content-rich interfaces. This data suggests that people prefer the “with countdown” and “with entertainment” formats. Participants favoured passenger animation icons under the “pointer icon” category, with some expressing that the “humanoid icon” appeared more graphic and conveyed the feeling of the passenger actively going to the pickup point.

Participants ranked their preferred kind among the three study indicators in the concluding subjective preference survey. The statistical findings aligned with the satisfaction statistics. Participants indicated that more interface information and “humanoid icon” alleviated their boredom and enhanced their engagement.

### Implications

#### Theoretical and practical implications

This study has theoretical and practical ramifications. First, the main theoretical contribution of this study is that it deepens and expands the research on waiting interface design in the field of human-computer interaction. This study uses the PAD three-dimensional emotion model in the special occupational group of online ride-hailing drivers to validate the applicability of this classical theory in mobile driving scenarios. It was found that the driver’s emotion and time perception were affected by different interface factors in the waiting situation, which provides an essential theoretical reference for subsequent research. Second, this study overcame the limitation of a single examination of interface elements in previous studies by measuring the main effects of the three elements and their interaction effects. Through the experimental design and data analysis, it was found that there was a significant interaction effect between the countdown indicator and the passenger animation icons in affecting perceived speed. This finding suggests that the influence of interface elements on users’ psychology is not a simple direct effect but rather a complex interaction. Specifically, in a countdown environment with explicit time information, anthropomorphic icons reinforce the user’s sense of control, thus speeding up time perception. In the absence of a time reference, pointer icons are more advantageous. This interaction effect complements the results of existing studies. It emphasizes the importance of fully considering the systematic interaction relationships between different interface elements when constructing future models of the user waiting experience. Finally, the multidimensional data collection metrics used in this experiment provide a new way of measuring user experience that integrates the user’s emotional experience, time perception, and subjective feedback, complementing the limitations of existing research.

Secondly, regarding practical consequences, our study offers four design recommendations highlighting interface variables designers should consider for influencing users’ time perception, emotional experience, satisfaction, and preference. The precise design recommendations are as follows:

1. A countdown indication’s existence markedly influenced participants’ user experience, as shown by the primary effect results across many dimensions of time perception, emotional responses, satisfaction, and preference. Consequently, future interface designers may use the findings of this study to enhance the user experience on various levels by incorporating a countdown indicator for the passenger’s anticipated arrival at the pickup point.
2. Passenger motion symbols demonstrate a significant advantage for user happiness and preference. While passenger animation icons do not influence users’ time estimates or emotional experience, most participants preferred “humanoid icon.” Consequently, designers may contemplate creating “humanoid icon” to enhance users’ preference for the interface subjectively.
3. The effects of the “with entertainment” type surpassed those of the “without entertainment” type in numerous aspects. This conclusion is largely congruent with the effects associated with the “with countdown” kind.
4. The countdown indication exhibited a notable interaction effect with passenger animation icons regarding the apparent speed in time perception. The “humanoid icon” was recognised considerably more quickly in the “with countdown” category, but the “pointer icon” was identified more swiftly in the “without countdown” category. Conversely, the “pointer icon” is recognised more rapidly in the “without a countdown” format. Notably, perceptions were often quicker for the “with countdown” variant.

This study’s conclusion can be a reference for application developers and user interface designers to scientifically optimise interface design aspects, thereby converting passive waiting into a tolerable interactive experience.

#### Positive impacts of the ride-hailing driver experience

The findings of this study hold direct and positive implications for enhancing the professional experience of online ride-hailing drivers. First, through measuring and analyzing multiple emotional dimensions among online ride-hailing drivers, the study reached reliable and authentic conclusions: optimizing the waiting interface design—such as providing a clear countdown indicator and moderate entertainment content—can effectively alleviate anxiety and boredom experienced by drivers during waiting periods. Second, this finding will enhance drivers’ emotional state during individual service trips in the short term, while fostering a stronger sense of belonging and job satisfaction toward the platform in the long run. Such human-centered design interventions are crucial against challenges such as high driver turnover and occupational burnout in the online ride-hailing industry. Finally, the recommendations proposed in this study offer valuable perspectives for optimizing waiting interface interaction design: the interface should be functional and caring. Preserving and prioritizing the social welfare of online ride-hailing drivers will foster driver stability and promote the industry’s sustainable development.

### Limitations and future work

This study validates the applicability of relevant theories while providing recommendations for optimizing the interface design during online ride-hailing drivers’ waiting periods; nonetheless, certain shortcomings persist that future research could address. The study did not investigate the individuals’ personal attributes, such as gender, age, and professional experience. Future research may examine the impact of these qualities by incorporating a broader spectrum of driver profiles, thereby offering a more thorough context. This study exclusively involved Chinese people engaged as online ride-hailing drivers in mainland China. In the future, we may simultaneously conduct research in many countries to examine the differences among online ride-hailing drivers across nations. Future research may explore and establish a broader range of icon categories, as this study investigated a restricted selection of experimental icons.

## Conclusions

This study examines the impact of various countdown indications, passenger animation icons, and entertainment fillers on time perception, emotional experience, satisfaction, and preference through comparative analysis. Theoretical foundations integrating time perception and the three-dimensional emotion model of PAD are utilised to examine the primary effects of interface design elements and their consequences for interaction. The trial findings indicate that a countdown indication should be incorporated into the waiting interface, along with entertaining material, since enhanced information during the waiting period markedly elevates the user experience and leads consumers to view the wait as shorter. The countdown indicator effectively enhanced the user’s sense of control, and when combined with the entertainment filler, it markedly alleviated the tedium of waiting. Moreover, humanistic and engaging icons are more likely to garner consumers’ preference during the waiting period for online ride-hailing drivers.

## Data Availability

All data generated or analysed during this study are included in this published article (and its Supplementary Information files).

## Declarations

### Statement

We have obtained informed consent from all subjects and/or their legal guardian(s) for the publication of identifying information/images in online open-access publications.
